# Current smoking behaviour among rural South African children: Ellisras Longitudinal Study

**DOI:** 10.1186/1471-2431-11-58

**Published:** 2011-06-23

**Authors:** Ramaijane J Mashita, Mahlapahlapana J Themane, Kotsedi D Monyeki, Han CG Kemper

**Affiliations:** 1 Department of Educational Studies, University of Limpopo, Sovenga, South Africa; 2 Chronic Disease of lifestyle unit, Medical Research Council, Tygerberg, 7505, South Africa; 3 VU University Medical Centre, The Institute for Health and Care Research (EMGO+), Amsterdam, the Netherlands

## Abstract

**Background:**

The use of tobacco products is the major cause of chronic diseases morbidity and mortality. Most smokers start the smoking habits from childhood and adolescent stages.

**Method:**

This was a cross-sectional study. A total of 1654 subjects (854 boys and 800 girls), aged 11 to 18 years, who were part of the Ellisras Longitudinal Study completed the questionnaire. Association between tobacco products use and habits, attitudes and beliefs were explored in this study. Logistic regression was used to estimate the association.

**Results:**

The prevalence of tobacco product use increases with increasing (4.9 to 17.1%) age among boys whereas girls do not smoke cigarette but only considerable number (1.0 to 4.1%) use home made tobacco products (pipe and snuff) among the Ellisras rural children. Parents and grand parents play a significant (about 50%) role in influencing smoking behaviour among the Ellisras rural children. Seeing actors smoking on TV shows was positively associated (p < 0.05) with smoking (OR = 2.3 95%CI (1.3-4.1 and OR = 2.3 95%CI 1.3-4.1 after adjusting for age and gender). However, women who smoke cigarette were significantly (p < 0.001) associated with success and success and wealth (OR = 2.8, 95% CI 1.5-5.2) even after adjusting for age and gender (OR = 2.9 95% CI 1.5-5.4).

**Conclusion:**

The usage of tobacco products was high among older boys. Girls did not smoke cigarette. This tobacco use behaviour mirrors the cultural norms and adult behaviour. The association of this tobacco used products with biological parameters will shed more light on the health of these children over time.

## Background

Tobacco use is a major public health concern, not only because of its indisputable impact on the health of both smokers and non-smokers, but because it is one of the preventable causes of morbidity and mortality. Smoking is a lifestyle factor that is strongly associated with certain forms of cancer [1-4] and cardiovascular diseases [2,5,6]. Internationally, public awareness and beliefs about the health risks of smoking have increased considerably, especially among adolescents and this was reflected in declining smoking rates in numerous developed countries [7]. However, in developing countries cigarette smoking is likely to increase tremendously in the next two decades as the tobacco industry concentrates its marketing on youth [8]. Indigenous knowledge, practice and traditional beliefs are sometimes also blamed for this escalating behaviour in the developing countries [9].

There is an increasing public awareness of health risks of tobacco products usage which is reflected in the declining rate of cigarette smoking in the developed countries, while in developing countries the rate is increasing [10-12]. For example, tobacco consumption, amongst adolescents in South Africa, that began in the 1960s, has peaked up in the last decade of the twentieth century [13]. In 1992, Martin et al. [7] reported that 31.5% of South Africans, 18 years and older smoked. This figure increased to 34% in 1995 which includes younger children who start smoking earlier [14]. Subsequently, Reddy et al. [15] reported that about one in five learners (21.1%) of Grades 8 to 11 in public schools were classified as current smokers nationally while the South African Demographic Health Survey [16] reported 3.3 to 7.0% for females. About 1.0 to 23.7% male adolescents aged 15 to 19 years old were reported to have ever use tobacco product daily.

This trend is indeed worrying especially with an increase of incidents of smokers among younger children of which little is known about their behaviour, beliefs and practices of tobacco products given the fact that these communities are traditionally known to be conservative. This study therefore, was intended to investigate the current smoking behaviour among rural South African children aged 11 to 18 years who are part of the ongoing Ellisras Longitudinal Study cross-sectionally.

## Methods

### Geographical area

Ellisras is a deep rural area situated within the north-western area of the Limpopo Province, South Africa. The population is about 50, 000 residing in 42 settlements [17]. These villages are approximately 70 km from the Ellisras town (23°40S 27°44W), now known as Lephalale, adjacent to Botswana border. The Iscor coal mine, Matimba and Medupi electricity power stations are the major sources of employment for many of the Ellisras residents, whereas the remaining workforce is involved in subsistence farming and cattle rearing, while the minority is in education and civil services. Unemployment, poverty and low-life expectancy seem to play a significant role in the rural South African population of which Ellisras rural area people are not an exception [18,19]. Schools in these villages are characterized by poor infra-structure, lack of teaching and learning resources (like laboratories and libraries) and lack of recreational and health facilities [20]. These schools are under-performing in terms of literacy and mathematics [21-23].

### Research design and sampling

The Ellisras Longitudinal Growth and Health Study (ELS) initially followed a cluster sampling method [24,25]. In brief, the study was undertaken at 22 schools (10 pre-schools and 12 primary schools). The selection was done randomly from 68 schools within the Ellisras area. Birth records were obtained from the principals of each school. Only those records that were verified against health clinic records were used to determine the age of each potential participant. Each of the 22 selected schools was assigned a grade with the expectation that most of the children in a particular age category (i.e. 3, 4,...9,10) would be found in that grade.

A total of 1654 children (854 boys and 800 girls) aged between 11 to 18 years who are part of the ELS participated in the study during the period 1 March to 3 May 2005 from the 1771 (923 boys and 848 girls) who participated in the anthropometric measurements of November 2003. The Ethics Committee of the South African Medical Research Council granted ethical approval prior to the survey and the parents or guardians provided informed consent. The children signed the assent form after receiving verbal assent from the project principal investigator.

### Data collection

The questionnaire that covers important concepts relating to tobacco products from Birth to Ten Study [26], the Amsterdam Growth and Health Longitudinal Study [27] and the first South African National Youth Risk Behaviour Survey [15] were studied. In these previous studies perceptions regarding teachers and other role models, who use tobacco products, attitudes based on exposure to the media and experimentation with tobacco products were thoroughly investigated. Lastly, the focus was directed at the South African Tobacco Control Legislation. Based on the definition of tobacco products, a list of concepts was developed. Questions addressing relevant concepts (knowledge, practice, attitudes, beliefs and advertisement) of the present study were extracted from the previously validated questionnaire. An expert panel was then convened to recommend which questions should be included in the knowledge, practices, attitudes and beliefs concepts. The principal investigator with the help of the Ellisras local teachers translated the questionnaire from English to the two local spoken languages (Northern Sotho and Setswana) and then translated back to English. The translation back to English showed no disparity with the Northern Sotho and Tswana languages (Ellisras rural area local language).

### Definition of concepts

A current smoker was defined as anyone who smoked at least one cigarette or any other type of tobacco product like pipe, snuff, home-made tobacco or indigenous tobacco per day at the time of survey. Those who never smoked a tobacco product at the time of the survey were considered non smokers. The attitude of the children's relatives and acquaintances towards cigarette smoking were measured indirectly by asking the children if their relatives and acquaintances allow people to smoke freely in their presence. In the interview, considerable participants were asked how strongly they believed in certain statements regarding smoking habits, attitudes and their beliefs on tobacco products. i) A packet contains ten cigarettes, ii) Box of BB or boxer weighs 12.5 g and iii) Snuff or powder-like tobacco product, Thekgwane or container carries 15 g of snuff.

### Quality control

All selected field workers underwent intensive training for one week prior to the survey. The inter tester (between fieldworkers) and intra tester (principal investigator and field workers) technical error of measurements ranged from 97 to 100% in agreement with the coding of the beliefs and attitude responses of the smoking questionnaire, whereas questionnaires relating to the knowledge of tobacco ranges from 95 to 98% agreement.

### Data analysis

All analyses were performed using the SPSS Version 14.0 (SPSS Inc., Chicago, IL, USA). This was a cross-sectional study. Analyses were run for descriptive statistics by gender and age. Chi-squared tests were used to compare sets of nominal data that had larger frequency counts, whereas the Fisher's exact test was used when frequency cells were small (less than five or ten) between genders. Logistic Regression analysis was applied to determine the association between tobacco products use as well as their beliefs and attitudes. The statistical significance was set at P < 0.05.

## Results

Table 1 presents prevalence of tobacco usage by age group and gender. The prevalence of cigarette smoking increased with increasing age (ranged from 4.9 to 17.1%) and was high among boys aged 11 to 18 years as compared to the usage of pipe (ranged from 1.4 to 3.9%) and snuff (1.0 to 3.9%) (Table 1). No Ellisras rural girls aged 11 to 18 years smoked cigarette. The prevalence of snuff usage ranged between 0.7 to 4.1% for girls aged 11 to 18 years. There was no significant difference between the prevalence of pipe and snuff usage between boys and girls through out the age range (Table 1).

**Table 1:** Prevalence of smoking by age group and gender for Ellisras rural children aged 11 to 18 years

Age range (years)	Boys (total N = 854)					Girls (total N = 800)				
	N	Cigarette	Pipe	Snuff	Total	N	Cigarette	Pipe	Snuff/ chew	Total
		(n) %	(n) %	(n) %	(n) %		(n) %	(n) %	(n) %	(n) %
11-12	142	(7)4.9	(2)1.4	(2)1.4	(11)7.8	134	0(0)	(1)0.7	(3)2.2	(4)3.0
13-14	205	(19)9.3	(6)2.9	(8)3.9	(33)16.1	196	0(0)	(2)1.0	(8)4.1	(10)5.1
15-16	308	(32)10.4	(12)3.9	(6)1.9	(50)16.2	294	0(0)	(4)1.4	(2)0.7	(6)2.0
17-18	199	(34)17.1	(7)3.5	(2)1.0	(43)21.6	176	0(0)	(4)2.3	(4)1.7	(8)4.5

Table 2 presents frequency and percentage frequency for positive responses on beliefs, habits and knowledge of home made usage of tobacco products for Ellisras rural children aged 11 to 18 years. The use of tobacco product among the Ellisras rural children family circle as reported by boys ranged from 5.7% to 24.9% and girls 0 to 27.8%. A considerable number of children (52% boys and 46% girls) were sent by somebody to buy smoking products in the local shop. Nine percent (9.0%) of boys and 5.8% girls believed they would use the tobacco product when they were adults.

**Table 2:** Frequencies and percentage frequencies for positive responds on believes, advertisement, habitual and knowledge of homemade use of tobacco product s for Ellisras rural children (boys = 854; girls = 800)

		Boys		Girls
		(n) %		(n) %
**Habits of Ellisras children of tobacco product**				
1. During the past seven days how many people use tobacco product at your home?				
	less than ten	more than ten	less than ten	More than ten
Smoked cigarette	(268)31.4	(5)0.6	(225)28.1	(4)0.5
Smoked tobacco	(256)30.0	(2)0.2	(216)27.0	(3)0.4
Use snuff	(193)22.6	(8)0.9	(179)22.4	(7)0.9
Chew tobacco	(5)0.6	0(0)	(2)0.3	0(0)
2. Does anyone send you to the shop to buy the following tobacco product for their own use				
Cigarette			(444)52.0	(368)46.0
Pipe tobacco			(223)26.1	(202)25.3
Snuff			(248)29.0	(238)29.8
Chew tobacco			(1)0.1	(4)0.5
3. Do the following people smoke cigarette or pipe? Your:				
Mother or Father			(213)24.9	(222)27.8
Grand parents			(189)22.1	(193)24.1
Brothers or sisters			(171)20.0	(157)19.6
Uncle or Aunts			(153)17.9	(144)18.0
Friends			(115)13.5	(98)12.3
**Believes of Ellisras children of tobacco product**				
Do you think it is acceptable				
1. For people to smoke in public places.			(140)16.4	(118)14.8
2. For people to chew tobacco in public places.			(178)20.8	(174)21.8
Do you believe that people				
3. Have the right to smoke where they please.			(77)9.0	(68)8.5
4. To chew or use tobacco anywhere they like.			(84)9.8	(67)8.4
5. Do you plan to use tobacco products when you are an adult?			(77)9.0	(46)5.8
**Knowledge of Ellisras children on home made tobacco product**				
1. Home made cigarette have the same effect on your health as the bought ones do			(243)28.5	(213)26.6
Home made cigarette do not have the same effect on your health as the bought ones do			(313)36.7	(328)41.0
There is no difference between homemade cigarette and bought ones			(298)34.9	(259)32.4
**Advertisement of Tobacco products on Television**				
How often do you see actors using tobacco product on Television or movie			(434) 50.8	(407)50.9
During the past 30 days how often do you see tobacco product brand or logo on Television			(366) 42.9	(311)38.9

There were relationships of various indicators of attitudes towards smoking with experimentation. For both boys and girls there was an effect on the perception of smoking when observing TV stars, men and women smoking. For example, almost similar number of boys and girls (20%) in Ellisras rural area admired TV stars and men who smoke cigarettes while women who used the tobacco products were less admired by many children (Table 3). Fifty point eight percent (50.8%) of boys and 50.9% of Ellisras rural girls watch Television actors in the movies using tobacco products (Table 2). There was no significant difference in the proportion of boys and girls who regarded men (19.7% vs 18.8% for cigarette, 15.2% vs 15.1% for snuff) and women (11.6% vs 13.4% for cigarette, 14.2 vs 15.6% for snuff) using tobacco products a symbols of being cool, rich and successful (Table 3).

**Table 3:** Frequencies and percentage frequencies for attitude of tobacco products for the Ellisras rural area children aged 11 to 18 years

	They look cool	Rich and successful	Stupid	Like losers	Very happy	No difference
	(n)%	(n)%	(n)%	(n)%	(n)%	(n)%
**Attitude of Ellisras rural children on Tobacco products**						
Boys						
1. TV star smoking cigarette.	(168)19.7	(56)6.6	(245)28.7	(189)22.1	(133)15.6	(63)7.4
2. Men smoking cigarette or pipe.	(164019.7	(51)6.0	(256)30.0	(238)27.9	(94)11.0	(51)6.0
3. Men chewing tobacco and using snuff.	(130)15.2	(29)3.4	(378)44.3	(196)23.0	(71)8.3	(50)5.0
4. Women smoking pipe or cigarette.	(99)11.6	(41)4.8	(403)47.2	(167)19.6	(86)10.1	(58)6.8
5. Women chewing tobacco or snuff.	(121)14.2	(14)4.8	(351)41.1	(196)23.0	(79)9.3	(66)7.7
Girls						
1. TV star smoking cigarette.	(153)19.1	(47)5.9	(254)31.8	(182)22.8	(84)10.5	(80)10.0
2. Men smoking cigarette or pipe.	(150)18.8	(57)7.1	(235)29.4	(218)27.3	(90011.3	(50)6.3
3. Men chewing tobacco and using snuff.	(121)15.1	(25)3.1	(360045.0	(177)22.1	97609.5	(41)5.1
4. Women smoking pipe or cigarette.	(107)13.4	(35)4.4	(337)42.1	(202)25.3	(67)8.4	(52)6.1
5. Women chewing tobacco or snuff.	(125)15.6	(40)5.0	(327)40.9	(187)23.4	(66)8.3	(55)6.7

Table 4 presents odd ratio, 95% confidence interval for the association of tobacco product usage and habits and attitudes towards the tobacco product among the Ellisras rural children. There was a significant (p < 0.001 to 0.05) association between the tobacco products use and the success of TV stars (OR 2.3 95% CI 1.3; 3.4) and smoking women (OR = 2.8 95% CI 1.5; 5.2; OR 2.9 95% CI 1.5; 5.4) even after adjusting for age and gender. The use of tobacco products and the rights of using tobacco products were significantly associated (OR 2.32 95% CI 1.4; 3.8 and OR 2.31 95% CI 1.4; 3.9) even after adjusting for age and gender (Table 4).

**Table 4:** Odds ratio and 95% confidence interval for the association of tobacco products use, attitudes and habits of tobacco products use among Ellisras rural children aged 11 to 18 years

	Unadjusted						Adjusted for age and gender					
	They look cool	Rich and successful	stupid	Like losers	Very happy	No difference	They look cool	Rich and successful	stupid	Like losers	Very happy	No difference
	OR (95%CI)	OR (95%CI)	OR (95%CI)	OR (95%CI)	OR (95%CI)	OR (95%CI)	OR (95%CI)	OR (95%CI)	OR (95%CI)	OR (95%CI)	OR (95%CI)	OR (95%CI)
**Attitude of Ellisras rural children on Tobacco products**												
TVSS	1.2 (0.7;1.8)	2.3* (1.3;4.1)	0.8 (0.5;1.2)	0.8 (0.5;1.3)	1.2 (0.7;1.9)	0.8 (0.4;1.6)	1.2 (0.7;1.8)	2.3* (1.3;4.1)	0.8 (0.5;1.2)	0.8 (0.5;1.3)	1.0 (0.6;1.7)	0.8 (0.4;1.7)
MS	0.9 (0.5;1.4)	1.5 (0.8;2.8)	0.7 (0.4;1.0)	1.2 (0.8;1.8)	1.1 (0.6;1.8)	1.6 (0.9;3.1)	0.9 (0.5;1.4)	1.6 (0.8;3.0)	0.6* (0.4;1.0)	1.2 (0.8;1.7)	1.1 (0.6;1.9)	1.7 (0.9;3.3)
MCT	1.4 (0.9;2.2)	1.2 (0.5;3.1)	0.8 (0.6;1.2)	1.0 (0.6;1.5)	0.6 (0.3;1.2)	1.9* (1.0;3.5)	1.4 (0.9;2.2)	1.2 (0.5;3.2)	0.8 (0.6;1.2)	1.0 (0.6;1.5)	0.6 (0.3;1.3)	1.8 (0.9;3.4)
WS	1.1 (0.7;1.9)	2.8* (1.5;5.2)	0.7 (0.5;1.0)	0.9 (0.6;1.4)	1.2 (0.7;2.2)	1.0 (0.5;2.1)	1.2 (0.7;2.1)	2.9** (1.5;5.4)	0.6* (0.4;0.9)	1.0 (0.6;1.6)	1.2 (0.6;2.1)	1.0 (0.5;2.1)
WCT	0.6 (0.3;1.1)	1.5 (0.7;3.1)	1.1 (0.7;1.5)	0.7 (0.4;1.1)	1.7* (1.0;2.8)	1.5 (0.8;2.7)	0.7 (0.4;1.2)	1.6 (0.8;3.3)	1.1 (0.7;1.5)	0.7( 0.4;1.1)	1.6 (0.9;2.7)	1.4 (0.8;2.6)
**Habits of Ellisras children of tobacco products**												
						Unadjusted			Adjusted for age and gender			
						OR (CI)			OR (CI)			
For people to smoke in public places.						1.17(0.7;1.9)			1.15(0.7;1.9)			
For people to chew tobacco in public places.						0.82(0.5;1.3)			0.82(0.5;1.3)			
Do you believe that people have the right to smoke where they please						2.32**(1.4;3.8)			2.31**(1.4;3.9)			
Do you believe that people have to chew or use tobacco anywhere they like.						1.44(0.8;2.5)			1.4(0.8;2.4)			
Do you plan to use tobacco products when you are an adult?						2.33**(1.4;4.0)			2.08**(1.2;3.6)			

* = p < 0.05; ** p < 0.001; OR = odds ratio, CI = 95% Confidence interval, TVSS = Television star smoking, MS = Men smoking, MCT = Men chewing tobacco, WS = Women smoking, WCT = women chewing tobacco

**Figure 1:**
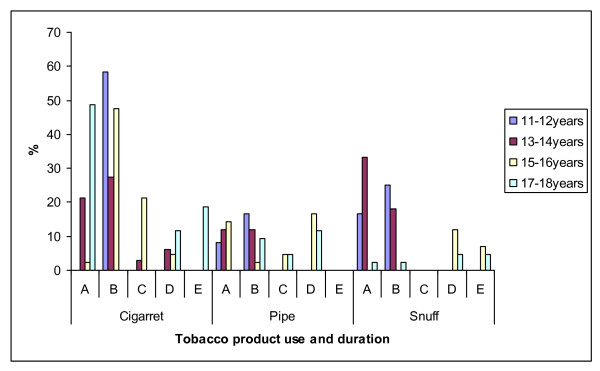
**The estimated period during which the Ellisras rural boys first start to use tobacco product**.

**Figure 2:**
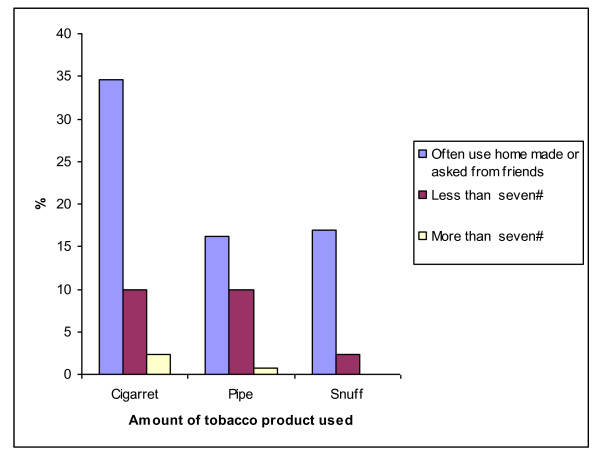
**The amount of tobacco product use by Ellisras rural boys age 11 to 18 year**.

**Figure 3:**
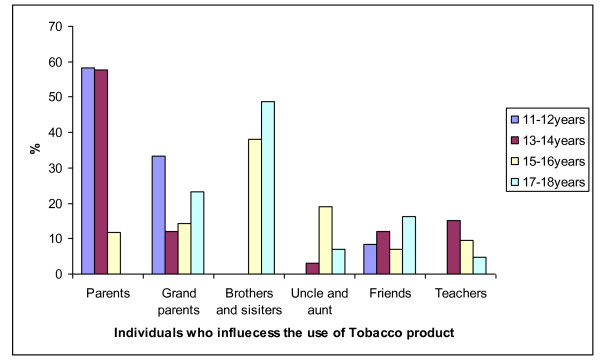
**Who influence the Ellisras rural boys to start using the tobacco products**.

Figure 1 shows the estimated period during which rural boys first started to use tobacco products by age group. The majority of children started smoking around the age of seven years. Figure 2 presents the amount of tobacco products used by Ellisras rural boys aged 11 to 18 years. The majority of participants (cigarette = 34.6%; pipe = 16.2%; snuff = 16.9%) did not know the amount of tobacco they used per week as they relied on others or use home made tobacco product while few children (10%) reported to use less than seven packets of cigarettes or seven grams of BB or boxer a week. Figure 3 presents the influential people in the use of tobacco product among the Ellisras rural children by age group. The majority (almost 50%) of younger children (11-14 years) were influenced by their parents and grand parents whereas teachers and friends also contributed in influencing (20%) older children (15-18 years).

## Discussions

The aim of the study was to evaluate habits, beliefs and practices of Ellisras rural children regarding tobacco products usage. The overall results indicated an increase in the prevalence (about 17%) of cigarette smoking by boys with age. Similar results were reported in South Africa nationally [15,16] on children of similar age groups. The usage of cigarette smoking by girls in this Ellisras population did not exist. This was different from the 14.9% girls grade 8 to 11 and 5.2 to 7.5% girls aged 15 to 19 years reported in South Africa nationally [15,16]. However, the prevalence of smoking among the Ellisras boys was lower compared to that of the Zambian boys (9.3%) whereas smoking among the Zambian girls was high (12.1%) as compared to findings of both the current study and the national survey in South Africa [15,16,28].

The children further reported that they did not admire women who smoke cigarette. Smoking initiations in Ellisras rural boys starts between aged 11 to 12 and increases with increasing age. Similar results were reported in Hong Kong, Global Youth Tobacco Survey Project, Zambia and Cape Town for children between the age of 11 to 18 years or [28-31]. However, Damas et al. [32] and Wen et al. [33] reported that most smokers had parents (n = 111, 57%), as well as friends who smoked (n = 187, 96.4%). Children who are surrounded by smoking friends and family members are more likely to be smokers [34,35]. Similar discoveries were found among the Ellisras rural children since the use of tobacco product by family members and visitors is a normal practice (more that 80% reported less than ten people using tobacco product at their home) (Table 2). In addition, there was a significant (p < 0.001) association (OR = 2.33 95% CI 1.4; 4.0 and OR 2.08, 95% CI 1.2; 3.6 after adjusting for age and gender) between tobacco products use and the possibility of using tobacco products as an adult by the Ellisras rural children.

Tobacco advertising bans have been effective in reducing consumption in a number of countries including South Africa, Zambia, New Zealand, Canada where the ban has been associated with the rapid decline in tobacco consumptions [28,36,37]. Yang et al. [38] reported that children admire their doctors when they smoke. However, the overall impact of tobacco advertisement by TV stars and some role models who use tobacco products openly in the community was two-fold in this Ellisras rural community. Firstly, it starts as a stimulant to viewers to start smoking, thus supporting the impression that it is a socially desirable activity. For example, the children in the current study significantly (p < 0.05) view TV stars who smoke as rich and successful (OR 2.3 95% CI 1.3;4.1 and OR 2.3 95% CI 1.3;4.1) after adjusting for age and gender). Zulu et al. [28] also reported a positive association between seeing a TV star smoking and the smoking behaviour among Zambian adolescents (OR = 1.90; 95% CI 1.26, 2.88). Secondly, though tobacco products cost increases yearly in South Africa, it seems to have resulted in the underestimation of its impact on tobacco use relative to a range of other public health issues. For example, in the current study, over 70% of the Ellisras children view home made cigarette as not having the same effect on one's health as the bought ones. Ultimately adolescents opted for the home made ones so as to imitate the TV stars, men and women who use tobacco in the Ellisras community.

Habits and beliefs in this Ellisras rural community appear to be central to the escalating use of tobacco products. In this study grand parents and parents were the most influential (50%) people to the children who smoke especially that the majority of the participants used home made or indigenous tobacco products (Figure 2). The possible explanation of this behaviour might be that boys in these communities spent most of their time outside their homes (engaged in animal husbandry and cattle rearing) and thus were exposed to people who use indigenous tobacco products as compared to their female counterparts (i.e. girls), who spent most of their time at home engaged in house chores, such as cooking, cleaning, fetching water and washing their family clothes, etc. On the one hand it is still a cultural taboo for a woman to smoke, let alone girls in Ellisras rural area. Similar trends are observable in Global Youth Tobacco Survey (GYTS) in countries that are still conservative like India and other parts of developing countries [39].

The South African Tobacco Product Control Act of 1993 and its amendments in 1999 and 2007 provides a hopeful paradigm shift in the use of tobacco products in the quest for the improvement of health and provision of awareness in communities about mortality emanating from chronic diseases. The findings of this study suggest that to succeed in these efforts, eradication of illiteracy should be the first hurdle to jump by health professionals in terms of providing primary health to these sectors of the community [40,41]. The South African population should make strides to shift from the traditional knowledge and medication, and seek new innovative ways of addressing issues facing the population with regard to health, climate, environment and lifestyle changes in line with the ongoing political transformation.

We acknowledge that we did not consider the socio-economic status of families of the participants in the analysis. Furthermore, our study is cross-sectional in nature. Many changes are taking place in South Africa today, which are of concern. These include the adoption of western diets, hence the changing of lifestyles which include among others, excessive use of contemporary tobacco products and an increase level of alcohol usage [42,43]. Also, a follow up on the use of tobacco products may shed more light on how smoking is related to other biological parameters in this population. Furthermore, the role of schools in the teaching of health education, to curb the escalating drug abuse in the South African schools will be helpful.

## Conclusions

The prevalence of tobacco use increase with increasing age among boys. Girls do not smoke but use snuff. Ellisras rural children do not admire women who smoke. The findings of this study have at least two implications for both public health and education authorities respectively. Firstly, for the public health sector, there is an urgent need to carry out intervention programmes that can boost primary health facilities to provide information regarding adverse effects in this community, something that includes the home made tobacco products usage. Secondly, for the education sector, there is also an urgent need to include health education in the curriculum to address the risk behaviour among children who smoke. For example, policy makers in education should begin to introduce life skills programmes that address this health hazard.

## Competing interests

The authors declare that they have no competing interests.

## Authors' contributions

RJM participated in the design, coordinated the data collection and critical revision of the manuscript for important intellectual content. MJT participated in the study design and critical revision of the manuscript for important intellectual content. KDM participated in the study design, data collection, analysis and interpretation of data, drafting of the manuscript, critical revision of the manuscript for important intellectual content and administrative, technical, and material support, such as supervision of the study. HCGK participated in study the design, data analysis, interpretation and critical revision of the manuscript for important intellectual content. All of the authors read and approved the final version of the manuscript.

## Pre-publication history

The pre-publication history for this paper can be accessed here:

http://www.biomedcentral.com/1471-2431/11/58/prepub
